# Modelling the impact and cost-effectiveness of the HIV intervention programme amongst commercial sex workers in Ahmedabad, Gujarat, India

**DOI:** 10.1186/1471-2458-7-195

**Published:** 2007-08-06

**Authors:** Isaac C-H Fung, Lorna Guinness, Peter Vickerman, Charlotte Watts, Gangadhar Vannela, Jagdish Vadhvana, Anna M Foss, Laxman Malodia, Meena Gandhi, Gaurang Jani

**Affiliations:** 1 MSc Control of Infectious Diseases, London School of Hygiene and Tropical Medicine, London, UK; 2 Health Policy Unit, Department of Public Health and Policy, London School of Hygiene and Tropical Medicine, London, UK; 3 Department of Chemistry, University of Massachusetts Lowell, Lowell, Massachusetts, USA; 4 Jyoti Sangh, Ahmedabad, Gujarat, India; 5 AIDS Control Society, Ahmedabad Municipal Corporation, Ahmedabad, Gujarat, India; 6 Resource Centre for Sexual Health and HIV/AIDS, New Delhi, India; 7 Department of Sociology, Gujarat University, Ahmedabad, Gujarat, India

## Abstract

**Background:**

Ahmedabad is an industrial city in Gujarat, India. In 2003, the HIV prevalence among commercial sex workers (CSWs) in Ahmedabad reached 13.0%. In response, the Jyoti Sangh HIV prevention programme for CSWs was initiated, which involves outreach, peer education, condom distribution, and free STD clinics. Two surveys were performed among CSWs in 1999 and 2003. This study estimates the cost-effectiveness of the Jyoti Sangh HIV prevention programme.

**Methods:**

A dynamic mathematical model was used with survey and intervention-specific data from Ahmedabad to estimate the HIV impact of the Jyoti Sangh project for the 51 months between the two CSW surveys. Uncertainty analysis was used to obtain different model fits to the HIV/STI epidemiological data, producing a range for the HIV impact of the project. Financial and economic costs of the intervention were estimated from the provider's perspective for the same time period. The cost per HIV-infection averted was estimated.

**Results:**

Over 51 months, projections suggest that the intervention averted 624 and 5,131 HIV cases among the CSWs and their clients, respectively. This equates to a 54% and 51% decrease in the HIV infections that would have occurred among the CSWs and clients without the intervention. In the absence of intervention, the model predicts that the HIV prevalence amongst the CSWs in 2003 would have been 26%, almost twice that with the intervention. Cost per HIV infection averted, excluding and including peer educator economic costs, was USD 59 and USD 98 respectively.

**Conclusion:**

This study demonstrated that targeted CSW interventions in India can be cost-effective, and highlights the importance of replicating this effort in other similar settings.

## Background

The HIV epidemic in India is no longer negligible, with an estimated 5.7 million people living with HIV in 2005 (0.91% of the adult population) [1]. Although the overall prevalence in India may be below 1%, in the southern states of Andhra Pradesh, Karnataka and Maharashtra, and the north-eastern states of Manipur and Nagaland, the ante-natal clinic prevalence is above 1% [2]. In Gujarat the prevalence is generally much lower (0.13% among ante-natal clinic attendees in 2004). However, in the city of Ahmedabad, the HIV prevalence in 2003 amongst ante-natal clinic attendees was 0.75%, and was 13.2% amongst CSWs [3,4]. This is particularly concerning since Ahmedabad is the seventh largest city in India with a population of over 4.5 million in 2005 [5].

Under guidance and support from the Ahmedabad Municipal Corporation AIDS Control Society (AMCACS), HIV prevention programmes in Ahmedabad started in 1997. Currently there are 26 operational HIV prevention projects among different sections of the society. One of these is the CSW prevention project run by Jyoti Sangh, a non-governmental organisation that promotes the welfare and empowerment of women [4]. This project is supported by the Department for International Development, UK, and is an integral part of the National AIDS Control Programme implemented across India under the guidance of NACO [4].

Jyoti Sangh has contact with CSWs from different areas of the city that work on the street and in other settings such as brothels and beauty parlours. Their four main strategies for reducing HIV transmission include: (1) Increase the knowledge of HIV/AIDS and sexually transmitted infections (STI) among CSW; (2) Improve the STI treatment of CSW and their clients; (3) Increase safer sex practices among CSW; and (4) Provide an environment that enables CSWs and the sex industry to promote safe sex behaviours. Outreach workers, assisted by peer educators, visit CSWs regularly to distribute condoms, promote safer sex practices and encourage CSWs to attend the free STI clinic. Using snowballing methods, Jyoti Sangh estimated that there are 4,000 (range 3,500–4,500) CSWs in Ahmedabad, and that since 1998, the number of CSWs reached by them has increased from 400 to over 3,050 in 2004.

In order to evaluate the impact of the Jyoti Sangh HIV prevention programme on sexual risk behaviour and prevalence of HIV and other STIs, two surveys were undertaken in August/September 1999 and November/December 2003. The surveys assessed the laboratory prevalence of *Chlamydia trachomtis*, *Neisseria gonorrhoea*, syphilis and *Trichomonas vaginalis*, and HIV, along with their behavioural correlates, such as condom usage and the number of clients per day. Over the 51 months between these surveys, Jyoti Sangh distributed over 5.5 million condoms. In addition, from January 2001 to December 2003, on average 2,342 CSWs were reached per month and 58 people were treated for STDs by the free clinic (range: 5 to 221 per month). Compared to the 1999 survey, the 2003 survey demonstrated a significant decrease in the prevalence of treatable STIs and a stabilisation of the HIV prevalence; this was reflected in a significant decrease in the reported number of sexual partners and a significant increase in consistent condom use [6].

This study aims to evaluate the Jyoti Sangh HIV intervention programme by using mathematical modelling and cost-effectiveness analysis with setting-specific epidemiological, behavioural and intervention data. The impact of the intervention is estimated in terms of HIV cases averted amongst CSW and their clients, and its cost-effectiveness as Indian rupees (INR) and US dollars (USD) per HIV case averted.

## Methods

Snow-balling (a gradual process through peer educators and outreach workers) was used to estimate the total number of CSWs in Ahmedabad for the two cross-sectional epidemiological and behavioural surveys undertaken among CSWs in August/September 1999 and November/December 2003. In the 1999 survey, a convenient sample of 314 CSWs was recruited from the 400–500 CSWs with whom Jyoti Sangh had rapport through outreach workers and peer educators. In the 2003 survey, 385 CSWs were sampled from a sampling frame of 1011 out of 3000 who expressed their willingness to participate in the study. The sample size was decided using a power calculation of 95% confidence intervals (CI) and 80% power based on the assumption that there would be at least 15% decrease in the prevalence of STIs among CSWs since the previous survey. A two-stage stratified cluster sampling was employed in which CSWs were stratified according to their work setting and sampling was carried out according to strata which represent the real proportion of different types of CSW in the population (Table A1) [4]. Data from these surveys were used to parameterise and fit a mathematical model to estimate the impact of the intervention over the 51 months between the mid-points of the two survey periods – the beginning of September 1999 to the end of November 2003. Mean ages of both surveys were similar (31.2 years in 1999 compared to 33 years in 2003), although the state of origin was slightly different and the proportions living with a husband or alone were slightly lower in 1999. A comparison of the designs (Table A1) and background characteristics of CSWs (Table A2) of the two surveys can be found in Appendix 1 [See Additional file 1] [4]. Cost data was also collected over this period so that the cost-effectiveness of the intervention could be estimated.

### The mathematical model

A deterministic mathematical model, Sex Work Gujarat 1.2 (Peter Vickerman and Charlotte Watts, LSHTM, UK) was used to assess the impact of the Jyoti Sangh intervention on HIV and STI transmission amongst CSWs in Ahmedabad. For this epidemiological setting, the model was used to estimate how changes in sexual behaviour and condom use and improvements in STI treatment, associated with being in contact with the intervention, may have reduced HIV and STI transmission.

Using behavioural and epidemiological data, the model divides the CSWs into sub-groups according to their level of condom use ('None of the time', 'Some of the time' and 'All of the time') and STI and HIV infection status. Established mathematical techniques [7] are used to estimate how HIV and a generic STI spread within the different CSW sub-groups and their clients over time with or without the intervention programme. By comparing these two scenarios, the relative decrease in HIV incidence and prevalence and the number of HIV infections averted by the intervention amongst CSWs and their clients can be estimated. The facilitation of HIV transmission by the presence of STIs in a sexual relationship [8,9] and the initial high viraemia stage of HIV [10] are incorporated into the model. The model equations are included in Appendix 2 [See Additional file 2].

### Data for mathematical model

The model was parameterised with behavioural data from the two surveys undertaken amongst CSWs by Jyoti Sangh, intervention-specific output data and data from the scientific literature. There were no data collected from clients, and so behavioural data for the clients were obtained from national surveys of paying clients of CSWs undertaken in 2001 by NACO [11]. There were no HIV or STI epidemiological data for the clients as they were notoriously difficult to collect data from. Because of this, the model's projections of the clients' STI and HIV prevalence amongst clients were not included as part of the fitting regime, and so were not restricted by any observed data. The different data used to parameterise the model are described below and are shown in Table 1.

**Table 1:** Model Input parameters and distribution types used in uncertainty analysis

Definition of model inputs	Model inputs (boundaries or *in italics: 95% CI*)	Probability Distribution	Date of behavioural survey and reference for model input values
***Coverage and Impact***			
Overall number of sex workers in Ahmedabad	4000 (3500–4500)	Triangular	Estimated by staff and peer educators of Jyoti Sangh through snow-balling method. Also personal communication with GJ on 7 July 2005.
Proportion of CSW recently reached by Jyoti Sangh intervention	0.586 (0.521–0.669)	Triangular	Average number of CSWs covered by the intervention program. Number covered/Total number = 2342/4000 = 0.5855 Max.: Number covered/min. total number = 2342/4500 = 0.5205 Min: Number covered/max. total number = 2342/3500 = 0.6692 (According to the routine data collected in monthly reports between Jan 2001 and Dec 2003)
Proportion of CSW reached using STD services per month	0.028 (0.028–0.041)	Uniform	Average of percentage of STD treatments (Female) in those CSWs covered by the programme who are believed to have STIs (Low estimate: 51.6%; High estimate: 75.5%; figures from [6] calculated from the percentage of CSW who do not have any STIs)
Average proportion of STDs treated effectively cured	0.8 (0.7–0.9)	Triangular	No data, treatment is assumed to be fairly effective.
Proportion reporting using condoms:			Intervention survey data from 1999 and 2003 [4]. 95% CI confidence intervals calculated using STATA.
*Before/Unreached:*			
*Never*	0.38 *(0.33–0.44)*	Normal	
*Sometimes*	0.29 *(0.24–0.34)*	Normal	
*Always*	0.33 *(0.27–0.37)*	Normal	
*After/Reached:*			
*Never*	0.047 *(0.028–0.073)*	Normal	
*Sometimes*	0.14 *(0.11–0.18)*	Normal	
*Always*	0.80 *(0.75–0.84)*	Normal	

***Epidemiological***			
Initial HIV prevalence amongst sex workers (first survey from 1999)	11.7% *(8.4%–15.9%)*	Normal	Survey data from 1999 [4] (Lower and upper estimates are 95% confidence intervals of the point estimates).
Average duration of generic STD included in model (months)			Varied in order to fit the STD prevalence in 1999 and 2003 surveys [6].
*CSW*	1 (0.5–1.5)	Uniform	
*CL*	1 (0.5–1.5)	Uniform	
Average duration of initial high infectivity phase (months)	1.5 (1–2)	Triangular	[10]
Average median duration between HIV infection and morbidity (months)	95	Constant	Median disease progression from HIV infection to AIDS takes 7.9 years in a cohort study in Mumbai, India. This was drawn from a truncated Weibull distribution [36].

***Behavioural***			
Average time span women sell sex (months)	(90–180)	Uniform	In the 2003 survey, average age of CSW = 33 years, average age when became CSW = 25.4 years [6]. Difference was used to estimate the lower bound, and the upper bound was set to twice the lower bound.
Average time span men buy sex (months)	(60–120)	Uniform	Median age (range) of CL is 28 (18,49); Median age at first sex with any commercial female partner (range) is 23 (10,40) [37]. Difference was used to estimate the lower bound, and the upper bound was set to twice the lower bound
Average number of clients per month per unreached CSW (using 1999 figure)	133 (119–157)	N/A	Average number of sexual partners per day during last month [6] and data from routine monthly reports between May 2002 and December 2003 were used to calculate an average.
Average number of clients per month per reached sex worker (using 2003 figure)	119 (107–140)	Triangular	Monthly reports of Jyoti Sangh from May 2002 to Dec 2003.
Average additional number of clients per month per unreached CSW	14 (11.6–17.0)	Uniform	Difference between the two figures above, calculated using Solver, using percentages from the two surveys [6].
Number of sex acts between one client and one sex worker in one encounter	1	--	No data. It is assumed to be near one, and that after discussion with GJ it was decided to be one.
Average number of CSWs seen by clients per month	(1–8)	Uniform	NACO survey reported median number of commercial female partners seen by a client in the last 3 months is 6 (1,27) [37]. However, high estimates of this parameter result in very high STI prevalences and so a lower range was used for modeling.
Proportion of time condom used, corresponding to:			
*"None of the time"*	0 (0–0.2)	Triangular	Zero was chosen as point estimate as it is the conservative estimation based on the definition "None of the time".
*"Some of the time"*	0.5 (0.2–0.7)	Triangular	Question framed in the questionnaire is 2 or 3 out of 5. So mean of 40% and 60% is 50% [6].
*"All of the time"*	0.8 (0.7–1)	Triangular	Question framed in the questionnaire is > 3 out of 5. So mean of 60% and 100% is 80% [6].

***Transmission Probabilities***			
Transmission probability of HIV per sex act:			[12, 14, 38]
*Male to Female*	0.002 (0.001–0.003)	Uniform	
*Female to Male*	0.001 (0.0005–0.003)	N/A	
Ratio of transmission probability: (Female to Male)/(Male to Female)	0.5 (0.5–1)	Triangular	
Transmission probability of generic STD per sex act:			[13, 15-20]
*Male to Female*	0.25 (0.1–0.5)	Triangular	
*Female to Male*	0.25 (0.1–0.5)	Triangular	
STD cofactor effect per sex act	3.1 (1.2–5)	Triangular	[8, 9]
Multiplicative cofactor during high viraemia phase of HIV infection	15 (10–20)	Triangular	[21, 22]
Proportion of time condom used that provides protection	0.85 (0.8–0.9)	Triangular	[23, 24]

CSW: commercial sex workers. GJ: Prof. Gaurang Jani. STI: sexually transmitted infection. STD: sexually transmitted disease. Note: If a certain value was calculated from a population sample, then a normal distribution was used and the range is the 95% confidence interval. If it was not produced from a population sample, then the range is the proposed uncertainty in the parameter used for the model uncertainty analysis, and if it was thought that one value was more likely then a triangle distribution was used. If no information existed on which value was more likely, then a non-informative uniform distribution was used.

#### Epidemiological data

Data on HIV and STI prevalence were reported in the two surveys. The HIV prevalence amongst surveyed CSWs was 11.7% (95% CI: 8.4–15.9%) in 1999 and 13.2% (95% CI: 10.1–16.9%) in 2003. The prevalence of different STIs is shown in Table 2, including details of the tests used. There has been a significant reduction in the overall prevalence of treatable STIs amongst CSWs, with 75.5% (95% CI: 70.3–80.1%) of CSWs having at least one STI in 1999 whereas in 2003, this had reduced to 51.6% (95% CI: 46.57%–56.78%). Because the model only simulates the transmission of one generic STI it was decided that it would be fit to the overall prevalence of STIs amongst CSWs in 1999 and 2003. Although this simplifies the nature of STI transmission, it was done to mimic the overall effect of treatable STIs on HIV transmission before and after the intervention. The model did not simulate the transmission of Herpes Simplex Virus-2 (HSV-2) because of a lack of epidemiological data. However, it was hoped that the effect of HSV-2 on HIV transmission was incorporated in a crude way within the HIV transmission probability at baseline through the uncertainty that was attached to this model parameter. It was assumed that this effect remained constant over the duration of the evaluation.

**Table 2:** Prevalence of different sexually transmitted infections and HIV among CSWs in two cross-sectional surveys in 1999 and 2003 [6]

STI	1999 N = 314	Prevalence (%) (95%CI)	2003 N = 385	Prevalence (%) (95%CI)
Trichomonas†	128	40.8 (35.3–46.4)	106	27.5 (23.1–32.3)
Gonorrhoea‡	60	19.1 (14.9–23.9)	23	6.0 (3.8–8.8)
Chlamydia*	53	16.9 (12.9–21.5)	37	9.6 (6.9–13.0)
Syphilis RPR	76	24.2 (19.6–29.3)	67	17.4 (13.5–21.6)
Syphilis TPHA	130	41.4 (35.0–47.1)	155	40.3 (35.3–45.4)

	N = 314		N = 401	

HIV**	37	11.7 (8.4–15.9)	53	13.2 (10.1–16.9)

CSWs: commercial sex workers † Wet Mount Microscopy and culture were used on vaginal swab sample ‡ Gram stain, culture in modified Thayer Martin (MTM)/modified New York culture (MNYC) media and antibiotic susceptibility test (and in 1999, PAGE2 as well) were used on endocervical swab sample. * PACE-2 test (and in 1999, Enzyme Immunoassay as well) were used on endocervical sample ** Double Enzyme-Linked ImmunoSorbent Assay (ELISA) test was used on serum sample. RPR: Rapid plasma regain. TPHA: Treponema Pallidum Haemagglutination test.

#### Behavioural data

Data regarding condom use and other sexual behaviour were obtained through self-reporting from the two intervention surveys. In addition, data from the Jyoti Sangh monthly monitoring reports were also used to estimate the number of clients per CSW per month.

The monitoring reports gave monthly estimates for the average number of sexual intercourses each CSW had per day for May 2002 to December 2003. By assuming one coital act per client, this data suggested that each CSW had on average 119 (range: 107 and 140) clients per month over this period. This was used for the CSWs reached by the intervention.

Because monitoring reports were not collected before 2002, and because only categorical data was collected in the behavioural surveys (proportion of CSWs that had less than 2, between 2 and 5 and over 5 clients per day), the client frequency for CSWs not reached by the intervention had to be estimated in other ways. This was done by using the monitoring data from 2002–2003 to calibrate the distribution data from the 2003 intervention survey, so replacing the categorical ranges of clients per day with point estimates. These point estimates were then used to calibrate the distributional data from the 1999 survey, and so produce estimates for the average client frequency per CSW not reached by the intervention – 133 (range: 119–157) clients per month. For details, see Appendix 3 [see Additional file 3].

#### Coverage of STD services

The number of CSWs reached and STI cases treated by the clinic per month were obtained from the monthly reports of Jyoti Sangh for January 2001 to December 2003. Monthly reports for earlier years are no longer available. The proportion of CSWs reached using STD services per month was calculated by dividing the monthly estimates of STIs treated with the estimated number of CSWs reached by the intervention in that month that had at least one STD. This was estimated by multiplying the average number of CSWs reached each month (from the monthly reports) by the overall prevalence of curable STIs from the 1999 (75.5%) or 2003 (51.6%) survey. These high and low estimates for each month were then averaged to give bounds for the proportion of CSWs reached using STD services per month.

#### Inputs from literature

The transmission probabilities for HIV [12-14] and the generic STD included in the model [13,15-20], STD cofactor effects per sex act [8,9], multiplicative factor during high infectivity phase [21,22] and proportion of time condom used that provides protection [23,24] were obtained from the literature.

### Model uncertainty analysis

Since there was uncertainty in the data used to parameterise the model (Table 1), an uncertainty analysis was conducted to obtain different model fits to available HIV and STI epidemiological data. This was done to assess the impact of parameter uncertainty upon the model impact estimates. Latin Hypercube Sampling, a type of stratified Monte Carlo sampling, was used to randomly generate over 10,000 different parameter sets, from the given parameter ranges (Table 1) [25]. In Latin Hypercube Sampling, probability distribution functions are defined for each parameter and the uncertainty intervals for each parameter are split into N strips of equal probability, where N is the number of runs undertaken in the uncertainty analysis. For each run, an interval is randomly chosen without replacement from each parameter uncertainty range, and a parameter value is randomly chosen from within the interval [25]. The actual number of parameter sets sampled was chosen to give sufficient model fits that generated HIV and STI prevalences within the 95 % CI of the CSW HIV prevalence in 2003 and STI prevalence in 1999 and 2003. The uncertainty range of the results was defined as the range within which 95% of the model fits lie. A simple linear regression analysis was performed on the different model fits to explore if there were any associations between the input parameters and different outputs (SPSS 12.0.1). To take into account the variability in the uncertainty of different inputs, standardised regression coefficients were used to measure the strength of association, and p-values were given by t-tests.

### Costing methods

Cost collection was based on the Costing Guidelines for HIV Prevention Strategies [26] and used an ingredients based methodology. Total provider costs were collected retrospectively. Financial and economic costs of the intervention were estimated for the period between the two surveys. Financial costs represent actual expenditure on goods and services purchased whereas economic costs reflect the opportunity cost of the project and also include the market value of goods or services that were not fully paid for, such as volunteer time and/or donated goods. Costs were classified as recurrent (personnel, supplies, transport, building maintenance) or capital (training, start-up costs, buildings, vehicles) and by category of programme input.

#### Input identification – Recurrent costs

Personnel records and staff interviews were used to identify staff and volunteers working on the project. Records of peer educator and other volunteer numbers, expenditures on medical and non-medical supplies as well as daily transport, building operating and maintenance costs (including communications) were obtained from project monitoring documentation and accounts. Additional transportation and communication expenses met by project staff were identified from interviews with personnel. Other recurrent items include expenses for meetings, workshops and special events (subsistence, accommodation etc), planning meeting costs, exposure visit costs, bank fees and support to people living with HIV/AIDS.

#### Input identification – Capital costs

Although the project began before our evaluation period, interviews with staff indicated that the 1999 baseline survey was key to recruiting CSWs and peer educators to the project. For that reason, and in the absence of earlier start-up data, the costs of the survey were estimated from project accounts and included as a start-up cost for the project. Staff training is a core activity of Jyoti Sangh. For training activities based in Gujarat, costs were calculated based on the number of participants, the number of days attended and the estimated cost per person day for an AMCACS training workshop. For other training activities, costs were obtained from expenditure records and interviews with staff.

Buildings used by the project were identified through interviews with staff and project records. Although the project does not own any vehicles, it was found that many staff use their own vehicles. Inventories were used to identify and estimate the prices paid for equipment used by the project (medical, audio-visual and furnishings).

#### Input valuation – Financial costs

Financial costs were valued as actual expenditure by the project on the identified recurrent and capital items over the period of the analysis. Capital costs were annualised using straight line depreciation assuming a project life of 10 years.

#### Input valuation – Economic costs

In obtaining the economic cost of personnel, full-time staff were assumed to work 100% of their time on the project, whereas volunteer staff were interviewed to ascertain their time spent on the project. The value of their time was based on the wages they could command in the local market. The value of volunteer time was estimated through interviews with a sample of seven peer educators and staff. The average time spent on the project by each peer educator and their average monthly income was calculated from these interviews, and was used to estimate the annual economic cost of all peer educators. The effect of the uncertainty in this calculation is explored using the extreme values (minimum and maximum) obtained for the peer educators' value.

Records did not identify the actual quantity of drugs or other supplies used and there were no major donations of these items, so for these inputs the economic cost was considered equivalent to the financial cost. The economic costs of transport, building maintenance and communication and other recurrent items were also valued using project expenditure. In the case of transport and communication, additional costs, incurred by staff out of their own pocket, were estimated from staff interviews and included in the calculation. To minimise recall bias, these costs were obtained for the most recent year of activity through interviews and extrapolated to earlier years.

The value of training and start-up items were valued at their financial cost. The 2004 rental value of the properties was used to estimate the economic value of buildings. Vehicles owned by staff have been included as economic costs by valuing them at the replacement value of a Bajaj scooter (the most common form of transport among staff) and assuming that 25% of their usage is related to project activities. The uncertainty around this value was explored by assuming that 10% to 50% of their usage is related to project activities. For the economic costs of other equipment, where possible, a local market price was obtained for the item. If this was not available the price paid was used.

Capital costs were annualised using an expected length of life ranging from 5–10 years, depending on the item, and the standard discount rate of 3% [27]. The effect of varying the discount rate from 3–10% was explored.

All costs were converted to constant 2004 prices based on the consumer price index [28] and summed to obtain an annual cost.

### Cost-effectiveness analysis

The cost-effectiveness ratio was defined as the change in cost over the change in effectiveness relative to having no intervention. In this case, the effectiveness was defined as the number of HIV infections averted and so the cost-effectiveness was the total costs divided by the total HIV infections averted between the first and second epidemiological surveys (51 months). The numbers of STIs averted were not included in the effectiveness measure because of the simplified nature in which the STIs were modelled. As no future costs and outcomes were included, no further discounting was carried out. Once the cost per HIV infection averted and been estimated, the cost per disability life year (DALY) gained was calculated using methods described in Fox-Rushby & Hanson (2001) [29]. The assumptions used in these calculations are described in Appendix 4 [see Additional file 4].

A two-way sensitivity analysis was carried out to test the robustness of the cost-effectiveness estimates to assumptions regarding key parameters. We used the extreme values of cost and impact to establish a range for the cost-effectiveness ratio.

This project was approved by the ethics committee of the Ahmedabad Municipal Corporation AIDS Control Society, Gujarat, India.

## Results

There were 119 runs that fit the survey data of HIV prevalence amongst CSWs in 2003 and STI prevalence amongst CSWs in 1999 and 2003. These model fits project that the intervention averted 624 (Uncertainty range: 310–1,191) HIV infections amongst CSWs in Ahmedabad from the beginning of September 1999 to the end of November 2003 (51 months) – 53.8% (Uncertainty range: 38.4%–68.8%) of the HIV infections that would have occurred without the intervention. In contrast, 5,131 (Uncertainty range: 2,282–8,896) HIV infections were averted among clients – 51.2% (Uncertainty range: 33.4%–64.1%) of those that would have occurred.

The model predicts that the intervention resulted in a reduction in the STI prevalence amongst the clients and commercial sex workers, which would have been stable otherwise. Indeed, if there had been no intervention, the HIV prevalence in December 2003 would have been 25.7% (Uncertainty range: 16.5%–39.4%) among CSWs and 3.1% (Uncertainty range: 1.4%–5.7%) among clients, nearly twice as high as the observed and projected HIV prevalences amongst CSWs and clients in 2003 with the intervention (1.6%, Uncertainty range: 0.9%–2.9% for clients). The model estimates that the STI prevalence of clients in the absence of intervention would have been 8.1% (Uncertainty range: 4.5%–12.2%), compared to 4.1% (Uncertainty range: 2.21%–4.35%) in the presence of intervention. Without the intervention, the model projects that the overall CSWs STI prevalence would have remained stable at 75.5%.

### Multi-linear regression analysis

Table 3 shows the standardised regression coefficients for the multi-linear regression analysis undertaken on the input and output of the uncertainty analysis. The main model parameters that had a significant impact upon the different impact outcomes of the intervention programme were the STD cofactor effect per sex act, proportion of CSWs recently reached, HIV transmission probability per sex act, average duration of STDs, and the average number of CSWs seen by each client per month. It is noted that the proportion of CSWs reached using STD services per month is not significantly associated with any of the outputs. This is because few infected CSWs attended the clinic each month (2.8%–4.1%) and so it had negligible effect. Probably for the same reason, the average proportion of STDs treated effectively cured is also not significantly associated with any of the outputs.

**Table 3:** Standardised regression coefficients of multi-linear regression model obtained from inputs and outputs of model uncertainty analysis.

**Standardised regression coefficient**	**Outputs of the mathematical model (dependent variables)**			
	
**Model input parameters (independent variables)**	**CSW HIV averted (%)**	**CSW HIV Prevalence (%) Difference**	**Client HIV averted (%)**	**Client HIV Prevalence (%) Difference**
**Overall number of CSW at any point in time**	-0.002	0.023	0.000	0.041
**Proportion of CSW recently reached by intervention**	0.566†	0.219†	0.520†	0.174†
**Proportion of CSW reached using STD services per month**	0.005	-0.004	0.003	0.020
**Average proportion of STDs treated effectively cured**	0.015	-0.020	0.004	-0.015
**Proportion of unreached CSW reporting using condoms**				
*None of the time*	0.415	0.215	0.259	0.092
*Some of the time*	0.245	0.096	0.107	0.036
*All of the time*	0.109	0.037	-0.029	-0.046
**Proportion of reached CSW reporting using condoms**				
*None of the time*	-0.280	0.233	-0.184	0.230
*Some of the time*	-0.258	0.655	-0.032	0.618
*All of the time*	-0.084	0.832	0.149	0.724
**Initial HIV prevalence of CSWs**	-0.272†	0.097	-0.220†	0.078
**Average duration of STDs (months)**				
*CSWs*	-0.049	-0.365†	-0.139*	-0.227**
*Clients*	-0.344†	-0.547†	-0.426†	-0.367†
**Average duration of initial high infectivity phase (months)**	0.129†	0.157†	0.154†	0.092*
**Average time span (months)**				
*CSWs sell sex*	-0.034	-0.003	-0.073**	-0.013
*Clients buy sex*	-0.068*	0.115**	0.036	0.122**
**Average no. of clients per month per CSW**				
*Unreached*	0.211**	0.182	0.138*	0.112
*Reached*	-0.242†	-0.153	-0.145*	-0.091
**Average number of CSW seen by one client per month**	0.012	0.442†	0.084	0.773†
**Proportion of time condom used, corresponding to**				
*"None of the time"*	-0.143†	-0.181†	-0.142†	-0.175†
*"Some of the time"*	-0.029	-0.069	0.024	-0.045
*"All of the time"*	0.392†	0.179†	0.275†	0.142**
**HIV Transmission probability per sex act**				
*Male to Female*	0.353†	0.692†	0.482†	0.211**
*Female to Male*	0.197†	0.661†	0.302†	0.696†
**STD Transmission probability per sex act**				
*Male to Female*	-0.129**	-0.271†	-0.180†	-0.136*
*Female to Male*	-0.172†	-0.220†	-0.200†	-0.125*
**STD cofactor effect per sex act**	0.604†	1.119†	0.727†	0.790†
**Multiplicative cofactor during high infectivity phase**	0.107†	0.168†	0.132†	0.133**
**Proportion of time condom used that provides protection**	0.283†	0.140**	0.226†	0.124*

* ≤ 0.05 ** ≤ 0.01 † < 0.001 CSWs: Commercial sex workers STD: sexually transmitted disease

### Cost analysis

The total economic and financial cost of the intervention, cost breakdown, and average annual costs are shown in Table 4. The total financial cost for the period of evaluation is USD 202,042 in 2004 currency. Economic costs are 2.7 times higher than the financial costs, reflecting the importance of the volunteer time, peer educators time and contributions from staff and peer educators in the form of communications and transport to the project. Personnel and peer educator costs are the most important project cost, and so the total costs are highly sensitive to assumptions made about the value of peer educator time. The average costs were relatively insensitive to variations in the other uncertain variables – vehicle costs and the discount rate.

**Table 4:** Total costs, cost breakdown, and average costs in constant 2004 Indian Rupees (INR) and American Dollars (USD)

**Cost Category**	**Financial**			**Economic**^~^		
	INR	USD	%	INR	USD	%
**Capital**						
Start up	61,186.29	1,328.98	0.7%	55,599.14	1,207.63	0.2%
Training	273,409.70	5,938.53	2.9%	247,149.71	5,368.15	1.0%
Buildings^8^	569,947.10	12,379.39	6.1%	1,873,552.80	40,694.02	7.2%
Vehicles	-	-	0.0%	20,216.74	439.11	0.1%
Medical equipment	18,108.58	393.32	0.2%	16,451.42	357.33	0.1%
Non-medical equipment	6,293.75	136.70	0.1%	5,719.04	124.22	0.0%
**Total Capital Costs**	928,945.41	20,176.92	10.0%	2,218,688.86	48,190.46	8.6%

**Recurrent**						
Personnel	3,442,371.55	74,769.15	37.0%	6,567,802.58	142,654.27	25.4%
Peer educators	1,309,599.26	28,444.81	14.1%	11,468,762.91	249,104.32	44.3%
Supplies – medical	168,979.54	3,670.28	1.8%	168,979.54	3,670.28	0.7%
Supplies – non medical	1,035,064.04	22,481.84	11.1%	1,035,064.04	22,481.84	4.0%
Transport	886,697.44	19,259.28	9.5%	2,214,647.44	48,102.68	8.6%
Building operating and maintenance	375,282.24	8,151.22	4.0%	1,066,256.73	23,159.36	4.1%
Other	1,155,068.08	25,088.36	12.4%	1,149,388.94	24,965.01	4.4%
**Total Recurrent Costs**	8,373,062.15	181,864.95	90.0%	23,670,902.19	514,137.75	91.4%
**TOTAL COSTS**	9,302,007.56	202,041.87	100.0%	25,889,591.04	562,328.22	100.0%

Average cost per CSW reached (assumes 2342 CSWs are reached*):						
Over evaluation period	3,971.82	86.27	-	11,054.48	240.11	-
Annualised	934.55	20.30	-	2,601.05	56.50	-

CSWs: Commercial sex workers. ^~ ^Financial and economic values for the capital items (except for buildings) were considered equal. However, costs were discounted in the economic valuation (3%) resulting in economic costs being lower than financial costs for these items. ^8^The economic value of buildings is based on local market survey. * estimated as in the impact model i.e. 0.78 of the estimated CSW population.

Using the average monthly number of CSWs reached from monthly records, the financial and economic cost per CSW reached per month are USD 86 and USD 240, respectively. The annual equivalents are USD 20 and USD 56, respectively.

### Cost-effectiveness analysis

The results of the cost-effectiveness analysis of this intervention programme are presented in Table 5 using 2004 currency. The cost-effectiveness ratio is presented with and without the peer educator economic costs because of the uncertainty around these estimates and to aid comparability with other studies that do not include these costs. The cost-effectiveness ratio ranges from USD 33.7–133.4 per HIV infection averted, when peer educator costs are valued at the financial cost. When the peer educator costs are included the ratio increases nearly two-fold, ranging from USD 55.6–218.5 per HIV infection averted. The costs per DALY saved range from USD 1.9 to 7.5 and USD 3.1 to 12.3, for the two scenarios, respectively.

**Table 5:** Cost-effectiveness analysis result (in constant year 2004 currency)

	*Peer educator cost valued as financial cost*	*Peer educator input valued as economic cost*
*No. of HIV infections averted among CSWs and clients (95% UR)*	*5,755 (2,592–10,087)*	
*Total cost (95% UR) (INR)*	*15,730,427 (15,653,389–15,918,536)*	*25,889,591 (25,812,552–26,077,699)*
*Cost per HIV infection averted (95% UR)*		
INR	*2,732.87 (1,552–6,141)*	*4,498.63 (2,559–10,060)*
USD	*59.37 (33.7–133.4)*	*97.71 (55.6–218.5)*
*Cost per DALY saved (95% UR)*		
INR	*153.95 (87–346)*	*253.38 (144–567)*
USD	*3.34 (1.9–7.5)*	*5.50 (3.1–12.3)*

CSWs: commercial sex workers INR: Indian rupees UR: Uncertainty Range USD: US Dollars Note: The exchange rate: INR46.04 = USD1.00 (average for the year 2004)

Source: [39]

## Discussion

This study used mathematical modelling and economic methods to evaluate the impact of the Jyoti Sangh HIV prevention project for CSWs in Ahmedabad. The project reached about three-quarters of the estimated CSW population in the city by the end of 2003 and involved three major components which have a direct impact on HIV transmission: promotion and distribution of condoms; free STD treatment; and counselling and behavioural change through peer education. For the period from the beginning of September 1999 to the end of November 2003 (51 months in total), the model projected that the intervention averted about half of the HIV infections that would have occurred amongst CSWs and clients in Ahmedabad without the intervention. The total number of HIV infections averted was estimated to be 5,755 (Uncertainty range: 2,548–10,140), and the cost-effectiveness ratio ranged from USD 38 to USD 133 per HIV infection averted when peer educator costs were excluded. When the peer educator costs were included the ratio ranged from USD 56 to USD 219.

Based on mathematical modelling, it has been suggested that sex worker interventions could drive the Indian HIV epidemic *"to extinction" *[30]. By assuming the proportion of unprotected sexual contacts reduces from 67% to 25% through condom promotion among CSWs and their clients, Nagelkerke *et al*. [30] predicted a fivefold decrease in the HIV prevalence in India after 30 years compared to if no intervention had occurred. For comparison, STI management alone, when assumed to result in a 30% reduction in HIV transmission, made the HIV prevalence decrease two- to threefold. Given these long-term impact predictions, it is important that effective interventions such as the Jyoti Sangh project are continued. Even though the model predicts that this intervention resulted in a 50% reduction in incident infections, there was still a modest increase in HIV prevalence observed from 1999 to 2003. This emphasizes the importance of using modelling to estimate the impact of HIV prevention projects and highlights that more may need to be done to reduce HIV incidence in this setting.

In a recent international initiative to evaluate the cost-effectiveness of various strategies to combat HIV/AIDS in developing countries, the cost per HIV infection averted of peer education and treatment of STI for CSWs in World Health Organisation South East Asia Region (Sear-D region), of which India is a member, was found to be 45, 47 and 50 international dollars (year 2000) given a coverage level of 50%, 80% and 95% respectively [31]. Our cost per HIV infection averted converts to the international dollar equivalent of 290 (excluding peer educator economic costs). The difference in these estimates can be attributed to differences in methodology to both costing and modelling of the impact. For example, Hogan *et al*. [31] will have produced much greater impact projections because they compared their intervention impact against the hypothetical 'no condom use' and 'no STI treatment' scenario, and estimated the impact amongst the general population. Also, it is unclear whether their cost estimates include the value of peer educator time. Indeed, rarely are these costs included in the costing of HIV prevention projects which maybe due to the different motivations of traditional volunteers who might forego earnings and peer educators who may actually acquire additional benefits from working in this manner. Together with the uncertainty in these values identified in this study, this lack of evidence calls for improved methods for understanding of peer educator time allocated to education activities and the value of their time to increase our confidence in the estimates and their impact on cost-effectiveness. However, the results compare favourably with interventions in sub-Saharan Africa, where the cost per DALY save for CSW peer education projects was estimated to be between 4.5 and 7.9 USD (2004 prices) [32]. In addition, the undiscounted lifetime costs of AIDS illness in India is approximately USD 1200 [33]. Thus given the limited access to HAART in India, if we assume no HAART, preventing the number of infections as predicted in the model would result in a cost saving of between USD 3.1 –12.1 million. With HAART, the savings would be much greater.

There is a less difference between the results from Jyoti Sangh and other studies using locally collected data. The results from 32 other HIV intervention programmes among CSWs in Tamil Nadu and Andhra Pradesh [34,35], reveal costs per CSW reached ranging from 5 USD to 50 USD. Also from these studies it is apparent that the programme in Ahmedabad had greater scale than most of its counterparts in the two southern states, with only three programmes in Andhra Pradesh reaching greater numbers of CSWs (3,847, 4,690 and 6,379 respectively in fiscal year 2002–2003) [35]. In contrast, the proportion of CSWs using STD services per month (2.8%–4.1%) in Ahmedabad was much lower than the number of CSWs/clients referred for STI treatment per CSW reached in any of the south Indian projects in one of the former studies [34]. However, this difference could be because a low proportion of those referred are using services. In addition, as noted in the results, this proportion was not significantly associated with the impact projections of the Jyoti Sangh project. Indeed, it is likely that unless there is a significant increase in the number of CSWs or clients attending the Jyoti Sangh STI clinics, the clinics will have negligible effect on HIV and STI transmission in Ahmedabad. Despite this, the clinics are still likely to provide a beneficial point of contact for CSWs and their clients.

Our mathematical model was limited to the sexual relationships between CSWs and their clients, and excluded any HIV and STI transmission to other partners. No attempt was made to estimate the impact of the intervention programme upon the general population beyond the clients, and so our impact projections are likely to underestimate the real impact of the project. In addition, the model simulated the transmission of all the STIs together and so did not separate cofactors for each STI. This may have reduced the accuracy of our projections. Limitations in data, especially amongst the clients, resulted in substantial uncertainty in our impact estimates. Improvements in routine data collection and management, will certainly improve the quality of future impact estimations.

We did not incorporate the evolving nature of the intervention programme into the model. In reality, the intervention programme has been increasing in coverage, and improving their service through feedback from peer educators and gaining experience through practice. This increases the uncertainty in our estimates of coverage parameters. However, the model uncertainty analysis and fitting procedures minimized the effects of this uncertainty on the model predictions.

There were some differences between the sampling methods used in the two surveys, and in the survey estimates for the state of origin of the CSWs, and the proportions living with a husband or alone (Table A2). They were not adjusted in the model, and were a common limitation of analyses that use data not collected for modelling purposes.

Another limitation of the analysis was that HSV-2 transmission was not modelled as discussed in the Methods. This was due to a lack of applicable HSV-2 prevalence data. However, it is unlikely that the intervention would substantially affect the prevalence of HSV-2 over 33 months because of its likely high prevalence in this population and the fact that it cannot be cured.

Recall bias in the two surveys is a limitation in this study, as it is hard to determine to what extent the data collected reflect reality except that the large drop in STI prevalence concords with the reported decreases in risk behaviour.

## Conclusion

This study demonstrated that targeted CSW interventions in India can be cost-effective, and highlights the importance of replicating this effort in other similar settings. This impact study can be used as a reference from which to compare the impact of other intervention programmes in India.

## Abbreviations

AMCACS: Ahmedabad Municipal Corporation AIDS Control Society

AIDS: Acquired Immune Deficiency Syndrome

CI: Confidence Interval

CSW: Commercial Sex Worker

DFID: Department for International Development, United Kingdom

HIV: Human Immunodeficiency Virus

INR: Indian Rupee

LSHTM: London School of Hygiene and Tropical Medicine

STD: Sexually Transmitted Disease

STI: Sexually Transmitted Infection

USD: US Dollar

## Competing interests

The author(s) declare that they have no competing interests.

## Authors' contributions

MG and LG conceived the idea of this cost-effectiveness modelling project. PV and CW wrote the mathematical model and its computer applications. JV administered Jyoti Sangh documentation and provided ICHF with the project data. ICHF collected, collated and analysed Jyoti Sangh data. ICHF applied the model to calculate the effect of the intervention under PV's supervision and with the help of AF. This was the MSc project of ICHF. GV collected the cost data from Jyoti Sangh and LG did the cost-effectiveness analysis. LM as the head of AMCACS supervises all HIV intervention projects in Ahmedabad. GJ is the senior advisor to the Jyoti Sangh CSW intervention project and contributed to the parameterisation of the model with his sociological insights. ICHF drafted the paper. PV, LG and AF revised it. PV, LG and ICHF wrote the appendices. All authors contributed to the final version of this paper and agreed upon its publication.

## Pre-publication history

The pre-publication history for this paper can be accessed here:



## Supplementary Material

Additional file 1 Appendix 1: Table A1 and Table A2. Table A1: Comparisons between the designs of the 1999 and 2003 surveys; Table A2: Background characterisitics of the commercial sex workers in the 1999 and 2003 surveys. Click here for file

Additional file 2 Appendix 2: Technical description of Sex Worker 1.2 model. It describes the mathematical model used in this study. Click here for file

Additional file 3 Appendix 3: Behaviour data calculation. More detailed description of the way the behaviour data was calculated. Click here for file

Additional file 4 Appendix 4: Assumption used in the calculation of the DALYs. Description of the assumption used in the calculation of the DALYs Click here for file

## References

[B1] UNAIDS (2006). 2006 Report on the Global AIDS Epidemic.

[B2] Report on the global AIDS epidemic. http://www.unaids.org/en/HIV_data/2006GlobalReport/default.asp.

[B3] National AIDS Control Organisation of India (2004). Annual Report 2002–04 (up to 31 July 2004).

[B4] Jyoti Sangh, Ahmedabad Municipal Corporation AIDS Control Society (2004). Prevalence and trend of Sexually Transmitted Infections and HIV among Female Sex Workers of Ahmedabad, Gujarat, India during 2000–2003 Executive Summary.

[B5] Mahatma Gandhi Labour Institute (Ahmedabad) (2004). Gujarat State Human Development Report 2004.

[B6] Jyoti Sangh, Sexual Health Resource Centre (2004). STI Prevalence Study.

[B7] Weinstein MC, Graham JD, Siegel JE, Fineberg HV, Turner C, Miller H, Moses L (1989). Cost-effectiveness analysis of AIDS prevention programs: concepts, complications and illustrations. Confronting AIDS: Sexual behaviour and intravenous drug use.

[B8] Korenromp EL, de Vlass SJ, Nagelkerke NJ, Habbema JD (2001). Estimating the magnitude of STD cofactor effects on HIV transmission: how well can it be done?. Sex Transm Dis.

[B9] Rottingen JA, Cameron DW, Garnett GP (2001). A systematic review of the epidemiologic interactions between classic sexually transmitted diseases and HIV: how much really is known?. Sex Transm Dis.

[B10] Pilcher CD, Tien HC, Eron JJ, Vernazza PL, Leu SY, Stewart PW, Goh LE, Cohen MS (2004). Brief but efficient: acute HIV infection and the sexual transmission of HIV. J Infect Dis.

[B11] National AIDS Control Organisation of India (2001). National Baseline High Risk and Bridge Population Behavioural Surveillance Survey.

[B12] European Study Group on Heterosexual Transmission of HIV (1992). Comparison of female to male and male to female transmission of HIV in 563 stable couples. European Study Group on Heterosexual Transmission of HIV. BMJ.

[B13] Quinn TC, Gaydos C, Shepherd M, Bobo L, Hook EW, Viscidi R, Rompalo A (1996). Epidemiologic and microbiologic correlates of Chlamydia trachomatis infection in sexual partnerships. Jama.

[B14] Royce RA, Sena A, Cates W, Cohen MS (1997). Sexual transmission of HIV. N Engl J Med.

[B15] Holmes KK, Johnson DW, Trostle HJ (1970). An estimate of the risk of men acquiring gonorrhea by sexual contact with infected females. Am J Epidemiol.

[B16] Hooper RR, Reynolds GH, Jones OG, Zaidi A, Wiesner PJ, Latimer KP, Lester A, Campbell AF, Harrison WO, Karney WW (1978). Cohort study of venereal disease. I: the risk of gonorrhea transmission from infected women to men. Am J Epidemiol.

[B17] Kretzschmar M, Welte R, van den Hoek A, Postma MJ (2001). Comparative model-based analysis of screening programs for Chlamydia trachomatis infections. Am J Epidemiol.

[B18] Lycke E, Lowhagen GB, Hallhagen G, Johannisson G, Ramstedt K (1980). The risk of transmission of genital Chlamydia trachomatis infection is less than that of genital Neisseria gonorrhoeae infection. Sex Transm Dis.

[B19] Mann JR, Stine CC, Vessey J (2002). The role of disease-specific infectivity and number of disease exposures on long-term effectiveness of the latex condom. Sex Transm Dis.

[B20] Ruijs GJ, Schut IK, Schirm J, Schroder FP (1988). Prevalence, incidence, and risk of acquiring urogenital gonococcal or chlamydial infection in prostitutes working in brothels. Genitourin Med.

[B21] Chakraborty H, Sen PK, Helms RW, Vernazza PL, Fiscus SA, Eron JJ, Patterson BK, Coombs RW, Krieger JN, Cohen MS (2001). Viral burden in genital secretions determines male-to-female sexual transmission of HIV-1: a probabilistic empiric model. AIDS.

[B22] Fideli US, Allen SA, Musonda R, Trask S, Hahn BH, Weiss H, Mulenga J, Kasolo F, Vermund SH, Aldrovandi GM (2001). Virologic and immunologic determinants of heterosexual transmission of human immunodeficiency virus type 1 in Africa. AIDS Res Hum Retroviruses.

[B23] Pinkerton SD, Abramson PR (1997). Effectiveness of condoms in preventing HIV transmission. Social Science and Medicine.

[B24] Pinkerton SD, Abramson PR, Turk ME (1998). Updated estimates of condom effectiveness. Journal of Association of Nurses in AIDS Care.

[B25] Blower SM, Dowlatabadi H (1994). Sensitivity and Uncertainty Analysis of Complex Models of Disease Transmission: an HIV Model, as an Example. International Statistical Review.

[B26] Kumaranayake L, Pepperall J, Goodman H, Mills A, Walker D (2000). Costing Guidelines for HIV prevention Strategies.

[B27] Drummond M, Stoddart GL, Torrance GW (1997). Methods for the Economic Evaluation of Health Care Programmes.

[B28] Reserve Bank of India (2003). Handbook of Statistics for India.

[B29] Fox-Rushby J, Hanson K (2001). Calculating and presenting disability adjusted life years (DALYs) in cost-effectiveness analysis. Health Policy and Planning.

[B30] Nagelkerke NJ, Jha P, de Vlas SJ, Korenromp EL, Moses S, Blanchard JF, Plummer FA (2002). Modelling HIV/AIDS epidemics in Botswana and India: impact of interventions to prevent transmission. Bull World Health Organ.

[B31] Hogan DR, Baltussen R, Hayashi C, Lauer JA, Salomon JA (2005). Cost effectiveness analysis of strategies to combat HIV/AIDS in developing countries. BMJ.

[B32] Creese A, Floyd K, Alban A, Guinness L (2002). Cost effectiveness of HIV/AIDS interventions in Africa: a review of the evidence. Lancet.

[B33] Over M, Heywood P, Gold J, Gupta I, Hira S, Marseille E (2004). HIV/AIDS Treatment and Prevention in India Modelling the Costs and Consequences.

[B34] Guinness L, Kumaranayake L, Rajaraman B, Sankaranarayanan G, Vannela G, Raghupathi P, George A (2005). Does scale matter? The costs of HIV-prevention interventions for commercial sex workers in India. Bull World Health Organ.

[B35] Dandona L, Sisodia P, Kumar SG, Ramesh YK, Kumar AA, Rao MC, Marseille E, Someshwar M, Marshall N, Kahn JG (2005). HIV prevention programmes for female sex workers in Andhra Pradesh, India: outputs, cost and efficiency. BMC Public Health.

[B36] Hira SK SHJ, Lanjewar DN (2003). The natural history of human immunodeficiency virus infection among adults in Mumbai. The National Medical Journal of India.

[B37] National AIDS Control Organisation (NACO) I (2001). National Baseline High Risk and Bridge Population Behavioural Surveillance Survey.

[B38] Quinn TC, Wawer MJ, Sewankambo N, Serwadda D, Li C, Wabwire-Mangen F, Meehan MO, Lutalo T, Gray RH (2000). Viral load and heterosexual transmission of human immunodeficiency virus type 1. Rakai Project Study Group. N Engl J Med.

[B39] FXHistory: historical currency exchange rates. http://www.oanda.com/convert/fxhistory.

