# Supplementary material for: Modelling the impact and cost-effectiveness of the HIV intervention programme amongst commercial sex workers in Ahmedabad, Gujarat, India
Source: BMC Public Health. 2007 Aug 6;7:195. doi: 10.1186/1471-2458-7-195 (PMC1999496; doi:10.1186/1471-2458-7-195)
Supplement: Additional file 1 — Appendix 1: Table A1 and Table A2. Table A1: Comparisons between the designs of the 1999 and 2003 surveys; Table A2: Background characterisitics of the commercial sex workers in the 1999 and 2003 surveys. [file 1471-2458-7-195-S1.pdf]

Appendix 1: Table A1 and Table A2

Table A1: Comparisons between the designs of the 1999 and 2003 surveys (adapted from [1])

	1999	2003
Total number of CSWs who have rapport with Jyoti Sangh	400-800 CSWs	2500-3500 CSWs
Study design	Cross-sectional survey	Cross-sectional survey
Sampling method	Convenience	Stratified Cluster*
Sampling frame (pool from which CSWs were recruited)	400-500 CSWs	1011 CSWs
No. of CSWs recruited	314	385 (125 women were tested in addition to the required sample)
No. of clinics run by Jyoti Sangh	1	3
No. of clinics used for data collection and their location†	1 – Relief Road	2- Wadj and Relief Road
Components of data collection	Behavioural data<br>Clinical data on STIs<br>Laboratory data on STIs/HIV	Behavioural data<br>Clinical data on STIs<br>Laboratory data on STIs/HIV

CSWs: Commercial Sex Workers * A more appropriate sampling method was adopted in the 2003 survey as the sampling universe, i.e. the number of CSWs Jyoti Sangh had rapport with, had grown since the 1999 survey. A two-stage stratified cluster sampling was employed in 2003. CSWs were categorised into 5 groups: Street CSWs who usually solicit sex on specific locations on the street (40%); Brothel based CSWs who work from lodges or brothels in groups of five or more (39%); Residential CSWs who work from home, often not full time CSWs (13%); Call girls who arrange work using their phones or mobile phones at varying locations (8%); and Mobile CSWs who are usually migrant CSWs staying in Ahmedabad for short periods of two to three months. Mobile CSWs were not included in the sample as they were less likely to have had contact with the outreach workers, making them difficult to locate and to follow up. † The clinics were the bases of the outreach workers and the nodes of the network of peer educators and the CSWs with whom they had rapport. Relief Road clinic was in the headquarter of Jyoti Sangh on Relief Road. A second clinic was opened later as a result of the development of the intervention programme.

Table A2: Background characteristics of the commercial sex workers in the 1999 and 2003 surveys (adapted from [1])

Variable	Percentage (n=314) (1999)	Percentage (n=385) (2003)
Age*		
18-25 years	29.3	19.2
26-35 years	44.6	42.3
36-45 years	22.6	33.0
46 years and above	2.8	5.5
State of origin		
Gujarat	60	70
West Bengal	21	15
Maharashtra	5	3
Others	14	12
With whom do you live currently?		
Alone	14.0	27.8
With husband	53.5	61.8
With employer/ in brothel	6.7	3.6
With peers, boyfriend (not in brothel)	8.9	3.4
Others/no response	16.8	0.3

* Mean ages at the 1999 and 2003 surveys were 31.2 years and 33 years respectively.

1. Jyoti Sangh, Ahmedabad Municipal Corporation AIDS Control Society: **Prevalence and trend of Sexually Transmitted Infections and HIV among Female Sex Workers of Ahmedabad, Gujarat, India during 2000-2003 Executive Summary**. Ahmedabad: Jyoti Sangh and Ahmedabad Municipal Corporation AIDS Control Society; 2004.
