# Supplementary material for: Modelling the impact and cost-effectiveness of the HIV intervention programme amongst commercial sex workers in Ahmedabad, Gujarat, India
Source: BMC Public Health. 2007 Aug 6;7:195. doi: 10.1186/1471-2458-7-195 (PMC1999496; doi:10.1186/1471-2458-7-195)
Supplement: Additional file 2 — Appendix 2: Technical description of Sex Worker 1.2 model. It describes the mathematical model used in this study. [file 1471-2458-7-195-S2.pdf]

Appendix 2: Technical description of Sex Worker 1.2 model

SexWork1.2 simulates the transmission of HIV resulting from heterosexual contact between sex workers and their male clients. The heterosexual transmission of one STD between these sub-groups is also simulated. The model is formulated in Borland C++ (Inprise Corporation, California, USA), as a set of deterministic ordinary differential equations that describe the movement of sex workers and their clients between discrete sub-populations – based upon their condom use and HIV infection status. These are described in turn below.

In the model the sex worker population is divided into three sub-groups with different levels of condom use ($i=0$, *1 and 2* for those that use condoms 'none of the time', 'some of the time' and 'all of the time' respectively). There is only one behavioural sub-group for the clients, and their condom use is determined by the sex worker. As with other HIV compartmental models, each behavioural sub-group of sex workers and clients is divided into those that are susceptible to HIV infection (x), those that are recently HIV infected and in the initial high viraemia phase (h), and those who have progressed into the low-viraemia phase (y). New individuals enter the susceptible population at a fixed per capita recruitment rate Λ_r where the subscript r determines the sex of individual ($r=0$ for clients and $r=1$ for sex workers). Susceptible individuals become infected with HIV at a per capita rate that is determined by the per-capita risk associated with their sexual behaviour (π_r).

When a susceptible individual becomes infected with HIV they are initially highly infectious (for an average period *1/ν*). They then enter a long period of low infectivity (average duration *1/δ*). In the model individuals remain in the population until they stop buying or selling sex (average duration $1/\sigma_r$), or until they cease having sex due to chronic HIV related illness (average duration *1/δ*). Equation 1 describes the HIV infection dynamics amongst the sex workers (r=1) or clients (r=0). It is important to note that the parameter *i* only has any meaning for the condom use of sex workers (r=1).

$$\begin{aligned}\frac{dx_{ri}}{dt} &= n_{ri}\Lambda_r - x_{ri}(\pi_r + \sigma_r)\\ \frac{dh_{ri}}{dt} &= \pi_r x_{ri} - h_{ri}(\sigma_r + \nu)\\ \frac{dy_{ri}}{dt} &= \nu h_{ri} - y_{ri}(\sigma_r + \delta)\end{aligned} \quad , \qquad \text{Equation 1}$$

where n_{ri} is the total number of clients (r=0) or the number of sex workers (r=1) in condom use sub-group *[r,i]*. The probability that a susceptible sex worker or client becomes HIV infected per unit time from unprotected sex (π_r) is one minus the probability of not getting infected over this time. The probability of not becoming infected per unit time is the product of the probabilities of not being HIV infected from any sexual act with their commercial partners. For clients, these partners can come from different condom use sub-groups. If c_r denotes the total number of sexual partners a client (r=0) or sex worker (r=1) has, then mathematically, the probability that a susceptible individual of sex r becomes HIV infected from their commercial sexual partnerships (π_r) per unit time is given by:

$$\pi_r = \begin{cases} 1 - \prod_{\forall i=0..2} (1-\varphi_{ri})^{C_r \rho_{ri}}, & if \quad r=0 \\ 1-(1-\varphi_{ri})^{C_r}, & if \quad r=1 \end{cases} , \qquad \text{Equation 2}$$

The function φ_{ri} is defined as the probability that a susceptible sex worker (r=1) or client (r=0) will become HIV infected per unit time from a particular commercial sexual partnership where the sex worker has a specific level of condom use (determined by i). For clients (r=0), the function $\rho_{ri,}$ defines the probability that a client buys sex from a sex worker with a particular level of condom use, and has the following form:

$$\rho_{ri} = \frac{n_{ri}}{\sum_{j=0..2} n_{rj}}, \qquad \text{Equation 3}$$

For clients (r=0), the product $c_r\rho_{ri}$ gives the total number of commercial sexual partnerships a client forms with sex workers in condom use group i.

The quantity φ_{ri} is the product of the probability that a particular client or sex worker is infected, and the probability that they transmit HIV to their susceptible partner over that time period (defined as D_{ri}). The subscript i defines the level of condom use of the sex worker in the partnership and r defines the sex of the susceptible partner.

The probabilities of HIV transmission between a susceptible and infected individual per unit time are derived from Weinstein *et al.* (1989). The probability (D_{ri}) that a susceptible individual (of sex r) becomes HIV infected if they have commercial sex with an HIV infected individual is dependent upon whether a condom is used; the per sex act HIV transmission probability when having unprotected sex with an HIV infected individual (β_r); and whether the infected partner is in the high or low viraemia phase of HIV (with high viraemia increasing the transmission probability by a cofactor α).

If in each unit of time, a susceptible sex worker (r=1, condom use sub-group *i*) or client (r=0, and their sex worker partner is from condom use sub-group i) has η sex acts with an infected sexual partner; f_i is the probability that a condom is used per sex act; *e* is the per sex act efficacy of the condom; β_r is the probability of HIV transmission per sex act from the opposite sex to sex *r*; and α is the extent to which STD co-infection of either partner increases the probability of HIV transmission, then D_{ri} can be written:

$$D_{ri} = 1 - \left[\left(1 - \frac{y^s_{r'i}}{n_{r'i}}\right)\left(1 - \frac{y^s_{ri}}{n_{ri}}\right) \frac{\left[\Gamma h_{r'i} + \Phi y_{r'i}\right]}{y_{r'i} + h_{r'i}} + \frac{\left[h_{r'i}\mathrm{H} + y_{r'i}\mathrm{E}\right]}{y_{r'i} + h_{r'i}} \left[1 - \left(1 - \frac{y^s_{r'i}}{n_{r'i}}\right)\left(1 - \frac{y^s_{ri}}{n_{ri}}\right)\right]\right],$$

Equation 4

where y^s is the number of individuals infected with an STD for different sub-groups *[r, i]*, and

$$\Phi = \left[1 - \beta_r \left(1 - f_i e\right)\right]^{\eta} \qquad \mathrm{E} = \left[1 - \alpha\ \beta_r \left(1 - f_i e\right)\right]^{\eta}$$
$$\mathrm{H} = \left[1 - \alpha\ \beta_r \left(1 - f_i e\right)\right]^{\eta} \qquad \Gamma = \left[1 - \alpha\ \beta_r \left(1 - f_i e\right)\right]^{\eta}$$

Equation 5

Here the variables *Φ*, *E*, *Γ* and *H* denote the probabilities that a susceptible person, who is having sex with an HIV infected person, does not become HIV infected per unit time in the following different situations; neither partner has an STD and the HIV infected partner is not in the high viraemia phase (*Φ*); at least one partner has an STD and the HIV infected partner is not in the high viraemia phase (*E*); neither partner has an STD but the HIV infected partner is in the high viraemia phase (*Γ*);at least one partner has an STD, and the HIV infected partner is in the high viraemia phase (*H*).

STD infection dynamics

The model simulates the transmission of one STD among sex workers and their clients. Both HIV susceptible and HIV infected individuals can acquire an STD. The per capita

probability of STD infection per unit time (πs_r) is analogous to the per capita probability of HIV infection per unit time (π_r) (*Equ. 2*). If the probability of STD transmission per sex act is βs_r, and y^s is the number of individuals infected with an STD, then for each partnership the probability that a susceptible individual (of sex r, sex worker is in condom use sub-group i) is infected with a STD per unit time (defined as D^s_{ri}) is:

$$D^s_{ri} = \frac{y^s_{r'i}}{n_{r'i}}\left(1 - \left[1 - \beta s_r\left(1 - f_i e\right)\right]^{\eta}\right), \qquad \text{Equation 6}$$

If x^s denotes the number of sex workers or clients susceptible to STD infection, then the transmission dynamics of the STD can be described using a set of deterministic differential equations (Equation 7). For this, all individuals are assumed to remain infected for a fixed period of time ($1/\mu_r$), and then become susceptible to STD infection once more.

$$\begin{aligned} \frac{d\,x^s_{ri}}{dt} &= n_{ri}\Lambda_r - x^s_{ri}\left(\pi s_r + \sigma_r + \frac{\delta\, y_{ri}}{n_{ri}}\right) + \mu_r\, y^s_{ri} \\ \frac{d\,y^s_{ri}}{dt} &= x^s_{ri}\,\pi s_r - y^s_{ri}\left(\mu_r + \sigma_r + \frac{\delta\, y_{ri}}{n_{ri}}\right) \end{aligned}, \qquad \text{Equation 7}$$
