# Supplementary material for: Modelling the impact and cost-effectiveness of the HIV intervention programme amongst commercial sex workers in Ahmedabad, Gujarat, India
Source: BMC Public Health. 2007 Aug 6;7:195. doi: 10.1186/1471-2458-7-195 (PMC1999496; doi:10.1186/1471-2458-7-195)
Supplement: Additional file 3 — Appendix 3: Behaviour data calculation. More detailed description of the way the behaviour data was calculated. [file 1471-2458-7-195-S3.pdf]

Appendix 3: Behaviour data calculation

Data from the intervention surveys gave estimates for the proportion of commercial sex workers (CSWs) that had <2 (27.1% in 1999 and 39.7% in 2003), between 2 and 5 (41.4% in 1999 and 36.9% in 2003) and >5 clients per day (30.2% in 1999 and 23.4% in 2003) in 1999 and 2003. The monitoring reports gave a mean, minimum and maximum monthly estimates for the average number of sexual intercourses each CSW had per day for May 2002 to December 2003. By assuming one coital act per client, this data projected that each CSW had on average 119 (range: 107 and 140) clients per month over this period. This was used as an estimate for the number of clients per month for a reached CSW.

To estimate the corresponding number of clients per month for unreached CSWs, a number of additional calculations had to be undertaken to calibrate the distribution data from the 1999 intervention survey. This was done by firstly replacing the ranges with unknown point variables (x, y and z where $x<2$, $2<y<5$ and $z>5$ clients per day) which were then multiplied by the distribution data to produce equations for the average number of clients seen by a CSW per day in 1999 and 2003. The equation for 2003 was then scaled up to per month and set equal to either the mean (119), maximum (140) or minimum (107) estimate for the number of clients per CSW per month from the routine data. With different starting values for x, y and z, the solver function in Microsoft Excel 2002 was then used to find different values of x, y and z that solved the equation for 2003. These values were then substituted into the equation for 1999 to produce estimates for the

number of clients per month for an unreached sex worker. This was repeated for different starting values. The minimum and maximum estimates produced using this method were used for the lower and upper bounds, whereas the point estimate was set to be the mid-point of the estimates produced when the 2003 equation was set equal to 119. This method estimated that unreached CSWs had 133 (range: 119-157) clients per month.
