# Supplementary material for: Modelling the impact and cost-effectiveness of the HIV intervention programme amongst commercial sex workers in Ahmedabad, Gujarat, India
Source: BMC Public Health. 2007 Aug 6;7:195. doi: 10.1186/1471-2458-7-195 (PMC1999496; doi:10.1186/1471-2458-7-195)
Supplement: Additional file 4 — Appendix 4: Assumption used in the calculation of the DALYs. Description of the assumption used in the calculation of the DALYs [file 1471-2458-7-195-S4.pdf]

Appendix 4: Assumption used in the calculation of the DALYs

The calculation of the DALYs uses the methodology described in [1]. These are crude estimates based on a scenario that a person will live for 20 years with the infection.

The following assumptions were used to estimate the DALY saved per HIV infection prevented:

Item	Assumed value	Source/ note
Age at death	45	[2] Low side estimate
Life expectancy at age of death	25	[2] Life expectancy at age of death assumed = life expectancy at birth
Discount rate	0.03	[1]
Age weight	0.04	[1]
Disability weight (HIV)	0.233	[2]
Duration of disability (HIV)	2 years	Assumption based on knowledge of disease progression [3]
Disability weight (AIDS)	0.505	[2]
Duration of disability (AIDS)	18 years	Assumption based on knowledge of disease progression [3]
Age at onset	25	Assumption

1. Fox-Rushby J, Hanson K: **Calculating and presenting disability adjusted life years (DALYs) in cost-effectiveness analysis**. *Health Policy and Planning* 2001, **16**:326-331.
2. Mathers CD, Lopez AD, Murray CJL: **The Burden of Disease and Mortality by Condition: Data, Methods, and Results for 2001**. In: *Global Burden of Disease and Risk Factors* Edited by Lopez A, Mathers C, Ezzati M, Jamison D, Murray C. New York: Oxford University Press.; 2006.
3. Bertozzi S, Padian N, Wegbreit J, DeMaria L, Feldman B, Gayle HG, J, Grant R, Isbell M: **HIV/AIDS Prevention and Treatment**. In: *Disease control priorities in developing countries* Edited by Jamison D, Breman J, Measham A, Alleyne G, Claeson M, Evans D, Jha P, Mills A, Musgrove P. New York: Oxford University Press; 2006.
